# A Norwegian cohort with STAT1-related disease – further expanding the clinical phenotype

**DOI:** 10.3389/fimmu.2025.1620291

**Published:** 2025-08-13

**Authors:** Karen Helene Bronken Martinsen, Torstein Øverland, Asbjørg Stray-Pedersen, Tore G. Abrahamsen, Børre Fevang, Hans Christian Erichsen Landsverk

**Affiliations:** ^1^ Division of Pediatric and Adolescent Medicine, Oslo University Hospital, Oslo, Norway; ^2^ Institute of Clinical Medicine, Faculty of Medicine, University of Oslo, Oslo, Norway; ^3^ Norwegian National Unit for Newborn Screening, Division of Pediatric and Adolescent Medicine, Oslo University Hospital Rikshospitalet, Oslo, Norway; ^4^ Section of Clinical Immunology and Infectious Diseases, Division of Specialized Medicine And Surgery, Oslo University Hospital, Oslo, Norway

**Keywords:** STAT1 GOF, STAT1 LOF, chronic mucocutaneous candidiasis (CMC), autoimmune hypertriglyceridemia, malignancy, ophthalmologic

## Abstract

**Purpose:**

Inborn errors of immunity (IEIs) caused by mutations in *STAT1* are associated with a broad range of clinical manifestations, ranging from relatively mild to life-threatening. Our aim was to give a clinical and molecular description of a Norwegian cohort with STAT1-related disease.

**Methods:**

This is a descriptive epidemiological study.

**Results:**

We present 23 patients with heterozygous *STAT1* mutations, from 12 unrelated Norwegian families. Eighteen individuals had *STAT1 gain-of-function* (GOF) variants. Chronic mucocutaneous candidiasis (CMC) was the most common manifestation, observed in 94% of patients. Herpesviruses caused morbidity in one-third of patients, with severe complications such as varicella meningitis, varicella retinitis and ulcerative CMV esophagitis seen in 17%. Autoimmune hypertriglyceridemia with GPIHBP1 autoantibodies was diagnosed in one patient, adding a new entity to STAT1 GOF. Fifty percent of patients suffered chronic ophthalmologic manifestations. Severe gastrointestinal manifestations were observed in 22% of patients. Five of the 23 patients had *STAT1 loss-of-function* (LOF) variants. Mendelian susceptibility to mycobacterial disease (MSMD) was detected in three patients. Malignancy and autoimmunity were observed in two patients, both were heterozygous for the p.Ala246Thr variant, which is likely associated with a more complex phenotype. Significant viral infections were also observed. Presently, our cohort represents the largest nationwide study on STAT1-related disease.

**Conclusion:**

We report novel clinical manifestations in STAT1 GOF, and suggest that heterozygous STAT1 LOF might be a more complex condition than originally presumed.

## 1 Introduction

Mutations in Signal transducer and activator of transcription 1 (*STAT1)* lead to various inborn errors of immunity (IEIs). More than 100 different mutations in *STAT1* located in all domains of the protein have been reported (1). Mutations can be transmitted in an autosomal dominant (AD) or autosomal recessive (AR) manner, and exert either a gain-of-function (GOF) or loss-of-function (LOF) effect (1). GOF variants are believed to result in enhanced STAT1-dependent responses to interferons (IFNs) I and II and interleukin-27 (IL-27) (2–4). LOF variants cause impaired STAT1-mediated signaling after stimulation with IFNs and ILs (5).

Chronic mucocutaneous candidiasis (CMC) has been known for almost 100 years (6) and is characterized by the persistent or recurrent presence of candida infections affecting the skin, nails and mucous membranes (7, 8). In 2011, AD STAT1 GOF was found to be the underlying genetic cause in approximately 50% of cases of AD CMC (8). Currently, more than 400 patients with STAT1 GOF have been described worldwide (3). STAT1 GOF has a broad range of phenotypes, ranging from relatively mild to life-threatening. The range of severity varies even within families with identical mutations. CMC is the hallmark symptom, described in 98% of cases. Patients also show increased rates of bacterial, viral and invasive fungal infections. More than one-third have autoimmune disease, most frequently affecting the thyroid gland. It is also associated with an increased prevalence of malignancy and vascular aneurysms. The presence of malignancy, invasive infections or aneurysms are considered negative prognostic factors, which decrease the cumulative survival of patients aged 60 years from 87% to 31% (7). Hematopoietic stem cell transplantation (HSCT) was previously associated with poor outcome and a mortality rate around 50% (9). The outcome after HSCT has however improved dramatically after the introduction of JAK (Janus kinase) inhibitors (JAKi) as a bridge-to-transplant treatment, with survival rates of around 90% according to recent reports (10).

STAT1 LOF is rarer than STAT1 GOF, with approximately 60 patients described (11). The phenotype depends on the mode of inheritance, with AR cases usually being more severe. AR STAT1 LOF can result in partial or complete STAT1 deficiency, with the complete form presenting at an earlier age and with more severe symptoms. Mendelian susceptibility to mycobacterial disease (MSMD) is the most frequent manifestation. A vulnerability to viruses belonging to the *Herpesviridae* is also seen, as well as bacterial infections and secondary hemophagocytic syndrome (12). AD STAT1 LOF is described as isolated MSMD (5, 11, 13).

## 2 Methods and materials

To date, the Norwegian patient population with a genetic STAT1 diagnosis has not been described. The primary aim of this study was to provide a detailed description of this cohort. Patients carrying a disease-causing, suspected or likely disease-causing *STAT1* variant were included in the study from January 2023 to April 2024.

Eligible patients were identified with help from members of the Norwegian network for primary immunodeficiency, a nationwide network for healthcare professionals involved in the treatment of patients with IEIs. We also collaborated with the departments of medical genetics with *STAT1* included in their targeted gene sequencing panels (Oslo University Hospital, Haukeland University Hospital and Telemark Hospital Trust) to identify patients.

Oslo University Hospital is a national competence center for patients with IEIs in Norway, and patients were invited to participate in the study during their regular follow-up appointment. Patients who did not have a follow-up in Oslo, were recruited via their local hospital.

Patients included in the study were asked to complete a detailed questionnaire. This was a non-standardized questionnaire based on previously described symptoms and manifestations of STAT1-related disease. The questionnaire was also designed to identify other affected family members.

As part of routine follow-up, blood samples which included various immunological parameters were drawn from the participants. In cases where a new blood sample was not feasible, previous results were evaluated.

The patients’ electronic records at Oslo University Hospital were analyzed. The records included documents sent from the patient’s local hospital or general practitioner.

The patients were considered to have STAT1 GOF or LOF based on a comprehensive evaluation of the genetic variant identified, clinical manifestations, family history, laboratory parameters, and currently available relevant literature. For all patients, clinical manifestations were assessed to determine whether they indicated GOF or LOF. Patients with known pathogenic variants in *STAT1* were classified accordingly. In cases where functional analysis of variants has been reported in the literature, the methodology of these studies was evaluated. If the methodology were found to be satisfactory, we used these results to interpret the functional effect of our variants. In case of previously unreported variants, blood samples for functional analysis were sent abroad as part of clinical investigations, and an evaluation of the patient’s symptoms was conducted to determine whether the variant indicated GOF or LOF. Samples for functional analysis were sent to the French National Institute for Health and Medical Research (Inserm) in Paris and the Advanced diagnostic unit at Freiburg University clinic.

Informed written consent was obtained from all patients or their legal guardians.

The study was approved by the Regional Committee for Health and Research Ethics in Norway.

## 3 Results

### 3.1 Patient population

We identified 24 patients from 12 nonrelated families, one of whom died at age 71, possibly due to cancer. All 23 surviving patients consented to participate in the study and are described here in detail. There was a male predominance, with 14 male patients.

Eighteen patients from nine families were classified as having STAT1 GOF. The patients were aged 5–57 years. Eleven patients (61%) were male. Debut of symptoms was early, with 67% (12/18) reporting symptoms within their first year of life.

Five patients from three families were classified as having AD STAT1 LOF. Three patients (60%) were male. The patients ranged in age from 8–54 years.

### 3.2 Genetic features

**Figure 1:**
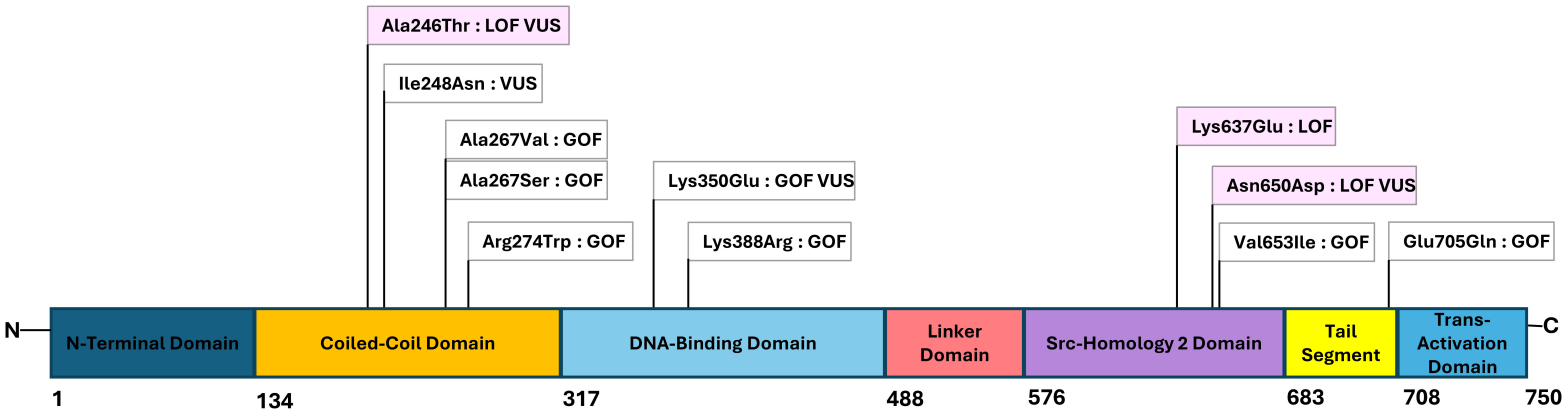
Figure with STAT1-mutations and their locations within the functional domains of the gene. The numbers below the figure indicating the amino acid number in the protein. Gain-of-function (GOF) and loss-of-function (LOF) variants are marked white and pink, respectively, and the novel variants of uncertain significance (VUS) noted.

Whole exome sequencing with targeted gene sequencing panels identified eleven different variants in *STAT1.* The variants were located in different domains of *STAT1*, with the coiled-coil domain (CCD) most frequently affected (45%) (**Figure 1**).

Eight variants were considered GOF and three were considered LOF. Individuals within the same family had identical genetic variants. The variant NM_007315.4(STAT1):c.820C>T, p.Arg274Trp was shared by two families (Family 2 and Family 12). Seven variants were regarded as pathogenic or likely pathogenic, and four missense variants were variants of uncertain significance (VUSs). Functional testing of these variants indicated either GOF or LOF, as shown in **Table 1**.

**Table 1:** The Norwegian families’ *STAT1* variants with predicted functional effects.

Family	Patients affected	cDNA change NM_007315.4	Predicted protein effect	Functional domain	Predicted functional effect	Reference
1	P1	c.2113G>C	p.(Glu705Gln)	Trans-activation domain	GOF	(33)
2	P2, P3, P4, P5, P6	c.820C>T	p.(Arg274Trp)	Coiled-coil domain	GOF	(34)
3	P7	c.1163A>G	p.(Lys388Arg)	DNA-binding domain	GOF	(35)
4	P8, P16	c.1048A>G	p.(Lys350Glu)	DNA-binding domain	Functional testing indicating GOF (Alanine scanning assay and pSTAT1 upon IFNα stimulation)	Personal communication with Dr. Anne Puel, 2019 and 2023
5	P9, P10	c.1948A>G	p.(Asn650Asp)	Src homology-2 domain	Presumed LOF due to absence of CMC. Reduced phosphorylation of STAT1 after stimulation with IFN’s	n/a
6	P11, P12, P19	c.743_744delinsAC	p.(Ile248Asn)	Coiled-coil domain	GOF	(36)
7	P13, P14, P21, P23^‡^	c.800C>T	p.(Ala267Val)	Coiled-coil domain	GOF	(37)
8	P15	c.799G>T	p.(Ala267Ser)	Coiled-coil domain	GOF	(8)
9	P17, P18	c.736G>A	p.(Ala246Thr)	Coiled-coil domain	LOF	(26)
10	P20	c.1957G>A	p.(Val653Ile)	Src homology-2 domain	GOF	(38)
11	P22	c.1909A>G	p.(Lys637Glu)	Src homology-2 domain	LOF	(39)
12	P24	c.820C>T	p.(Arg274Trp)	Coiled-coil domain	GOF	(34)

Table showing the families and the affected family members, and the different variants in *STAT1* and their predicted protein and functional effects. ‡, Deceased patient; GOF, Gain-of-function; LOF, Loss-of-function; CMC; Chronic mucocutaneous candidiasis; n/a, Not available.

**Figure 2:**
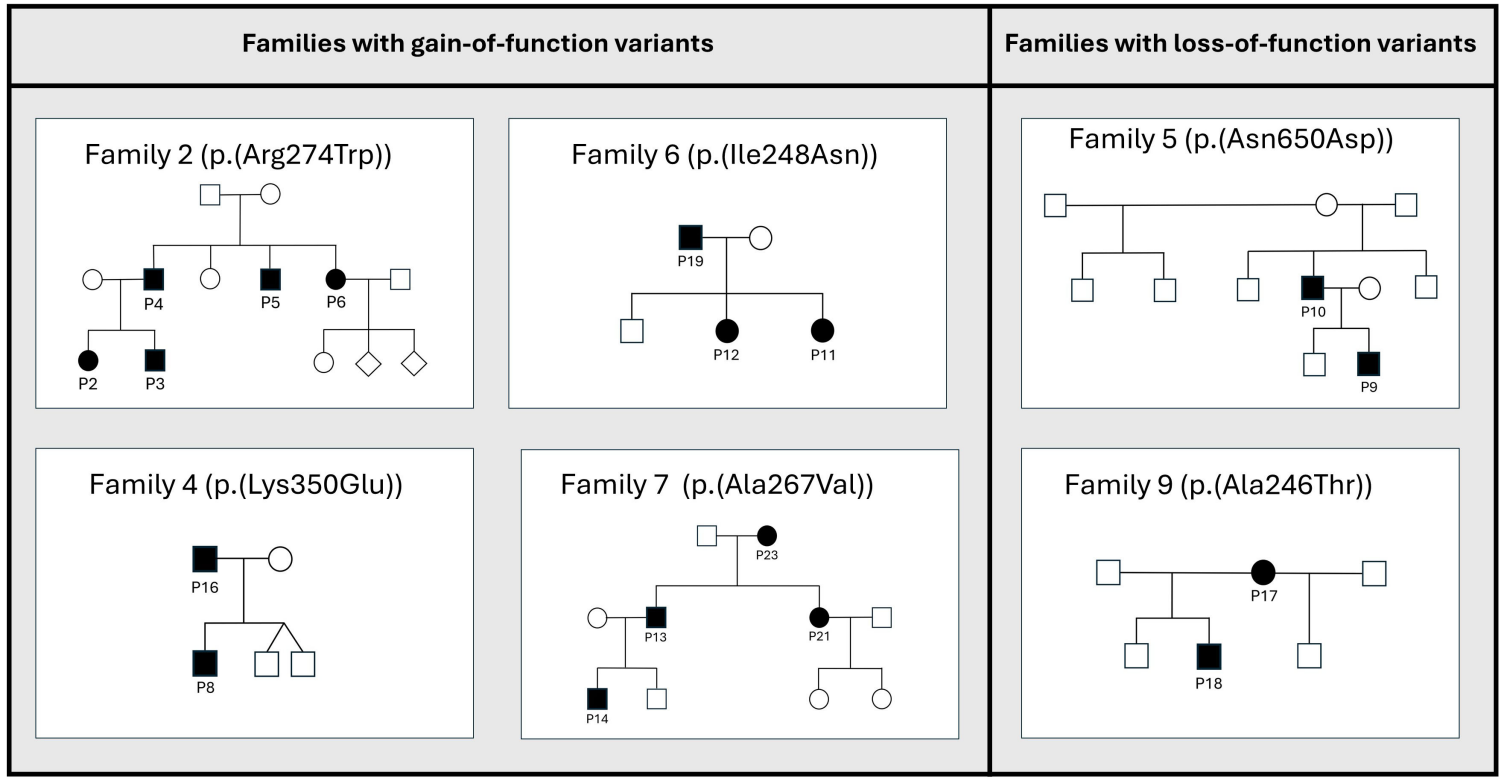
Pedigrees of the STAT1-families with several affected family members. Families with de novo variants, and only one affected member(i.e.families 1, 3, 8, 10, 11 and 12) are not shown in the figure. Affected members shown as black circles (females) or squares (males), unaffected members shown as white circles/squares. Diamond shape indicating unknown gender.

**Table 1** and **Figure 1** show an overview of the demographics and the molecular findings. **Figure 2** shows family pedigrees of the families with several affected family members.

### 3.3 Clinical manifestations in STAT1 GOF

#### 3.3.1 Infections

The patients had an increased infectious susceptibility, with CMC and recurrent respiratory tract infections being the most common manifestations. Infections in the eye and periorbital region affected 50% (9/18) of the patients. Bacterial skin infections were detected in 50% (9/18) of the patients.

**Table 2** summarizes the pathogens detected in the patients.

**Table 2:** Table showing the various pathogens detected in the STAT1-patients, with the specific patients in whom the pathogens were detected listed in brackets.

Verified microbe	Gain-of-function patients n=18	Loss-of-function patients n=5
Bacteria		
*Staphylococcus aureus*	8 (44%) (P1 - P2 - P6 - P7 - P8 - P13 - P16 - P20)	
*Streptococcus pneumoniae*	5 (28%) (P1 - P3 - P4 - P7 - P8)	
*Haemophilus influenza*	3 (17%) (P7 - P15 - P20)	
*Staphylococcus lugdunensis*	2 (11%) (P7 - P13)	
*Streptococcus pyogenes*	3 (17%) (P2 - P3 - P20)	
*Staphylococcus epidermidis*	1 (6%) (P7)	
*Streptococcus dysgalactiae*	1 (6%) (P13)	
*Propionibacterium acnes*	1 (6%) (P13)	
*Helicobacter pylori*	1 (6%) (P19)	
*Clostridium difficile*	1 (6%) (P1)	
*Mycoplasma pneumonia*	1 (6%) (P15)	
*Moraxella cataralis*	1 (6%) (P20)	
*Pseudomonas aeruginosa*	1 (6%) (P20)	
Viruses		
Varicella zoster	5 (28%) (P4 - P6 - P7 - P15 - P20)	2 (40%) (P9 - P17)
Influenza A & B	3 (17%) (P1 - P7 - P8)	2 (40%) (P9 - P10)
SARS-CoV	3 (17%) (P8 - P11 - P19)	3 (60%) (P9 - P10 - P18)
Metapneumovirus	2 (11%) (P7 - P8)	
Adenovirus	2 (11%) (P7 - P20)	1 (20%) (P9)
Parainfluenza	2 (11%) (P7 - P20)	
Epstein Barr virus	1 (6%) (P1)	1 (20%) (P9)
Cytomegalovirus	1 (6%) (P7)	2 (40%) (P9 - P10)
Respiratory syncytial virus	1 (6%) (P20)	1 (20%) (P9)
Coxsackie-virus		1 (20%) (P9)
Mycobacterias		
BCG		3 (60%) (P10 - P17 - P22)
Fungi		
*Candida albicans*	15 (83%) (P1 - P2 - P3 - P4 - P5 - P6 - P7 - P8 - P13 - P14 - P15 - P16 - P20 - P21 - P24)	
Dermatophytes	3 (17%) (P7 - P14 - P16)	
*Aspergillus fumigatus*	1 (6%) (P7)	
*Penicillium* spp.	2 (11%) (P7 - P8)	
Others		
Demodex mites	1 (6%) (P6)	

##### 3.3.1.1 Fungal infections

CMC was the most common infectious manifestation, observed in 94% (17/18). Debut of CMC was early, with 61% reporting fungal infections within their first year of life. *Candida albicans* was identified in 83% (15/17), and dermatophytes were identified in 18% (3/17). The oral cavity was affected in all of the patients suffering CMC (17/17), the esophagus and skin were affected in 59% (10/17). Onychomycosis affected 35% (6/17) and fungal scalp infections affected 12% (2/17) of the patients. Five out of seven female patients (71%) suffered recurrent vulvovaginal candidiasis, with debut in childhood/adolescence. Among the male patients, 36% (4/11) had genital fungal infections.

Two patients were treated for systemic fungal infections, caused by *Candida albicans* and *Aspergillus fumigatus*. *Penicillium* species were identified in two patients.

##### 3.3.1.2 Bacterial infections

Staphylococcal infections were most frequently identified (n=11), with *Staphylococcus aureus* being responsible for most documented infections (n=8). Streptococcal infections were documented in eight patients, with *Streptococcus pneumoniae* most commonly identified (n=5). One patient suffered severe necrotizing pneumonia caused by *S.pneumoniae*. Another patient contracted vision-threatening endophthalmitis due to *Propionibacterium acnes*.

##### 3.3.1.3 Viral infections

Herpesviruses were associated with morbidity, particularly due to reactivation of Varicella zoster virus (VZV). Herpes zoster was documented in 28% (5/18) of patients, with recurrent episodes in one patient. One patient had VZV retinitis complicated with acute retinal necrosis, and another experienced VZV meningitis. Notably, none of the patients had severe or prolonged course of primary VZV-infection. One patient suffered severe, treatment-resistant ulcerative cytomegalovirus (CMV) esophagitis. Epstein-Barr virus (EBV) was found in biopsies from the gastrointestinal tract on several occasions in one patient.

Two pediatric patients underwent reactive viral polyarthritis, one of which were secondary to adenovirus.

Warts were detected in 35%, and 12% of patients had molluscum contagiosum.

##### 3.3.1.4 Other infectious manifestations

One patient had a pustular skin infection with multiple Demodex mites within the pustules.

#### 3.3.2 Autoimmunity and inflammatory manifestations

Autoimmune and inflammatory manifestations were observed in 78% (14/18) of the patients, 93% of whom had multiple manifestations.

##### 3.3.2.1 Autoimmunity

Hypothyroidism was observed in 39% (7/18) of patients, and one patient had subclinical/latent hypothyroidism. Anti-thyroid antibodies were detected in 29% (2/7) of patients. Type 1 diabetes with positive anti-GAD antibodies was diagnosed in 6% (1/18), 11% (2/18) had DAT-positive autoimmune hemolytic anemia (AIHA), 6% (1/18) had seronegative rheumatoid arthritis, and 6% (1/18) had primary hyperparathyroidism.

One patient suffered from extreme hypertriglyceridemia. High titers of autoantibodies against GPIHBP1 (glycosylphosphatidylinositol-anchored high density lipoprotein binding protein-1) were found to be the underlying cause.

##### 3.3.2.2 Organ specific inflammatory manifestations

###### 3.3.2.2.1 Ophthalmologic manifestations

Chronic ophthalmologic manifestations were observed in 50% (9/18) of the patients; these manifestations were a combination of infectious and inflammatory. The patients suffered from chronic conjunctivitis, keratitis, blepharitis, chalazion, hordeolum and meibomian gland dysfunction. Vision-threatening manifestations were observed in 1/3 of the patients.

###### 3.3.2.2.2 Gastrointestinal manifestations

Gastrointestinal manifestations were observed in 72% (13/18) of patients, with abdominal pain being the most prominent symptom. Complicated manifestations such as substantial esophageal strictures and stenosis, ulcerative inflammatory bowel disease and eosinophilic esophagitis were observed in 22% (4/18) of patients.

###### 3.3.2.2.3 Respiratory tract manifestations

Chronic respiratory symptoms were observed in 56% (10/18) of patients, with chronic mucous production being the most common complaint (80%), followed by chronic cough (60%) and dyspnea (50%). Two patients were diagnosed with asthma. Bronchiectasis was found in 11% (2/18) of patients.

###### 3.3.2.2.4 Dermal manifestations

Chronic dermal manifestations were described in 83% (15/18), with eczema seen most frequently (33%, 6/18). Patients were also diagnosed with psoriasis, rosacea, urticarial rash and severe acne vulgaris.

###### 3.3.2.2.5 Vascular manifestations

Cerebral imaging was performed in 22% (4/18) of patients as a screening for cerebral aneurysms. No aneurysms were identified. Calcifications in the abdominal and thoracic aorta were described in one patient, and another had vasculitic histology changes in a biopsy from the genital area.

#### 3.3.3 Malignancy/premalignant manifestations

None of our patients were diagnosed with cancer. Premalignant conditions were seen in 39% (7/18), mainly affecting the gastrointestinal tract (in 6 out of 7), but also affecting the genital tract and hematological system.

**Table 3** provides a comprehensive overview of the clinical manifestations.

**Table 3:** Table showing clinical manifestations in each of the STAT1-patients.

Family number	Patient number	Sex	Age at inclusion	Infectious manifestations	Autoimmune/inflammatory manifestations	Other manifestations	Immunomodulating treatment
GOF-patients							
1 p.(Glu705Gln)	P1	F	20	CMC, respiratory tract infections, peritonsillar abscess, genital abscess, clostridium colitis, warts	Esophageal strictures, sterile esophagitis, inflammatory enteropathy, severe aphtous stomatitis, aphtous genitalis	Chronic musculoskeletal pain, hyperhidrosis	Tofacitinib, barcitinib, steroids, adalimumab, tacrolimus, colchicine, budesonide
2 p.(Arg274Trp)	P2	F	15	CMC, respiratory tract infections, skin infections, molluscum contagiosum	Hypothyroidism, blepharoconjunctivitis	Acne vulgaris	No
	P3	M	8	CMC, respiratory tract infections, molluscum contagiosum, virally induced polyarthritis	Blepharoconjunctivitis	Eczema	No
	P4	M	42	CMC, respiratory tract infections, herpes zoster, varicella retinitis, warts	Hypothyroidism, chronic mild duodenitis	Eczema	No
	P5	M	44	CMC, warts	Hypothyroidism, blepharoconjunctivitis, rosacea	Chronic headache and myalgia	Tofacitinib
	P6	F	35	CMC, herpes zoster, VZV meningitis, respiratory tract infections, demodicosis, deep seated abscesses groin	Hypothyroidism, AIHA, seronegative rheumatoid arthritis, AI hypertriglyceridemia with multiple episodes of acute pancreatitis, rosacea,	Seborrheic dermatitis, papulopustular rash, comedones, cholecystitis. Monoclonal B-cell lymphocytosis (MBL) of non-CLL (chronic lymphocytic leukemia) type.	Barcitinib, ruxolitinib, steroids, leflunomide, methotrexate, rituximab
3 p.(Lys388Arg)	P7	M	12	CMC, respiratory tract infections, necrotizing pneumonia, pulmonary aspergillus infection, CMV esophagitis, herpes zoster,	AIHA, anti-GAD positive diabetes mellitus type 1, inflammatory papillomatous changes in esophagus and vocal cords secondary to chronic CMV-infection	Hemiplegic migraine, calcifications abdominal and thoracic aorta, bronchiectasia	Steroids, Rituximab, immunoglobulins, Mycophenolate mofetil, Ruxolitinib. Stem cell transplantation: Conditioning with anti-thymocyte globuline (ATG, fludarabine, treosulphane and thiptepa. Graft-versus-host prohylaxis with cyclosporine and mycophenolate mofetil.
4 p.(Lys350Glu)	P8	M	16	CMC, respiratory tract infections, warts	Psoriasis, blepharoconjunctivitis and chalazion, oral lichen planus		No
	P16	M	47	CMC, respiratory tract infections, skin infections	Hypothyroidism, psoriasis, blepharitis/conjunctivitis		No
6 p.(Ile248Asn)	P11	F	9	CMC, respiratory tract infections, warts		ADHD, neurodevelopmental disturbance, asthma, discomfort fingertips	No
	P12	F	5	CMC, respiratory tract infections, skin infections		Delayed development (speech and fine motor skills), asthma, eczema, discomfort fingertips	No
	P19	M	43	H.pylori gastritis, respiratory tract infections	Gastritis	Peripheral neuropathy, sigmoid tubular adenoma with low-grade dysplastic changes, chronic back pain, eczema, allergy	No
7 p.(Ala267Val)	P13	M	57	CMC, systemic C.albicans infection, chronic skin infections with micro-abscesses, P.acnes endophtalmitis	Blepharoconjunctivitis, gastritis		No
	P14	M	33	CMC, skin infections,	Hypothyroidism, blepharoconjunctivitis	Growth retardation, delayed puberty, single GTK-attack, acne vulgaris	No
	P21	F	41	CMC, recurrent inguinal abscesses, genital HPV and molluscum	Esophageal strictures, esophageal ulcers, chronic sinusitis with reactive osteitis, cholecystolithiasis, rosacea, latent/subclinical hypothyroidism	Nodular colonic adenoma with low grade dysplasia, GERD, chronic diarrhea, lymphadenopathy liver hilum, cervical dysplasia, perioral and periorbital dermatitis, primary hyperparathyroidism	Barcitinib
8 (p.(Ala267Ser)	P15	M	30	CMC, respiratory tract infections, herpes zoster, warts		Bronchiectasis	No
10 p.(Val653Ile)	P20	M	11	CMC, respiratory tract infections, pneumonias, skin infections (abscesses), herpes zoster, reactive polyarthritis secondary to adenovirus-infection	Perioral and periorbital dermatitis, reactive polyarthritis, blepharitis, chalazion		No
12 p.(Arg274Trp)	P24	F	51	CMC	Hypothyroidism, esophageal stenosis with need of repeated endoscopic blocking, eosinophilic esophagitis	Migraine, cervical disc prolapse	No
LOF-patients							
5 p.(Asn650Asp)	P9	M	8	EBV and adenovirus-infection, chickenpox twice, herpes zoster, recurrent respiratory tract infections, gastroenteritis, molluscum contagiosum		Asthma, chronic GI-complaints, adenotonsillectomy, febrile seizures	No
	P10	M	38	BCGitis with skin ulcerations, disseminated BCG-infection with multiple skeletal lesions, hepatomegaly and lymphadenopathy. CMV encephalitis, warts, respiratory tract infections		Fatigue, unexplained childhood anemia, chronic GI-complaints/IBS, tubular cecal adenoma with low-grade dysplasia, ruptured Meckel diverticulum	No
9 p.(Ala246Thr)	P17	F	54	BCG-itis with granuloma formation, respiratory tract infections, paronychia, GI-infections, herpes zoster, warts, aseptic meningitis	Cicatricial pemphigoid, recurrent diverticulitis with sigmoid resection	Hodgkin lymphoma, asthma, severe urticaria, keloid scar formation, repeated sinus surgeries, tonsillectomy, repeated unexplained anaphylaxis	Steroids, methotrexate
	P18	M	29	Severe suppurative lymphadenitis in submandibular region (presumed atypical mycobacteria), respiratory tract infections, paronychia, fungal infections, conjunctivitis, warts	Psoriasis, granulomatous inflammation and lymphadenopathy	T-cell rich large B-cell lymphoma, asthma, hypogonadotrop hypogonadism, atrial septal defect, dilated cardiomyopathy, pilonidal cyst, severe caries, tonsillectomy	No
11 p.(Lys637Glu)	P22	F	30	Disseminated BCG-infection with dermal and extensive skeletal manifestations			No

The table includes a column for immunomodulating treatment in the patients where this was relevant.

### 3.4 Clinical manifestations in STAT1 LOF

#### 3.4.1 Infections

Increased rate of infections was reported by 80% (4/5), with debut at an early age. The patients suffered frequent respiratory tract infections (80%, 4/5), mycobacterial infections (60%, 3/5), gastrointestinal tract infections (40%, 2/5) and paronychia (40%, 2/5).

One patient had MSMD only, the remaining patients had complex phenotypes.

##### 3.4.1.1 Fungal infections

Onychomycosis since early childhood was reported in one patient. The same patient had substantial mucous membrane candidiasis under ongoing chemotherapy, which was more pronounced than normally seen.

##### 3.4.1.2 Viral infections

Morbidity due to viruses, mainly herpesviruses, was seen. This included CMV encephalitis in one patient and disseminated herpes zoster in another. One patient reported having chickenpox twice, followed by herpes zoster infection. This patient was also admitted to the hospital with primary EBV-infection and adenovirus.

Warts were reported by 60% (3/5), and 20% (1/5) had molluscum contagiosum.

##### 3.4.1.3 Mycobacterial infections

Complications secondary to Bacillus Calmette-Guerin (BCG) vaccination were observed in 60% (3/5). Two patients had disseminated BCG infection with extensive skeletal manifestations, and one patient developed a large granuloma. The patients were successfully treated with long-term tuberculostatica. The remaining two LOF patients were never BCG vaccinated.

One patient suffered severe suppurative lymphadenitis at five years of age which was suspected to be mycobacterial.

#### 3.4.2 Autoimmunity and inflammatory manifestations

Cicatricial pemphigoid affecting the oral cavity, conjunctiva and skin was documented in one patient. The same patient additionally had chronically elevated inflammatory markers, recurrent episodes of diverticulitis and keloid scar formation.

Another patient had recurrent episodes of granulomatous inflammation and lymphadenopathy of unknown cause.

#### 3.4.3 Malignancy

Hematological malignancy was documented in 40% (2/5) of patients. One patient was diagnosed with histiocyte/T-cell rich large B-cell lymphoma at the age of 26 years. The other patient was diagnosed with nodular lymphocyte rich Hodgkin lymphoma at the age of 30 years, and suffered a late recurrence of the malignancy at 55 years of age. The two patients shared the same genetic variant (p.Ala246Thr).

#### 3.4.4 Other manifestations

One patient was diagnosed with atrial septum defect and later developed dilated cardiomyopathy. One patient had a ruptured Meckel diverticulum.

**Table 3** provides a comprehensive overview of the manifestations in the patients.

### 3.5 Immunological investigations

All patients underwent extensive immunological investigations. A blood sample was obtained at the time of inclusion in 78% (18/23) of the patients. Previous laboratory values of interest were also evaluated. The patients had from one to more than 20 lymphocyte subset quantifications performed, with a median of six measurements per patient for STAT1 GOF and a median of two measurements per patient for the LOF patients. T- and B-cell subpopulations were analyzed in all but one patient, with a median of two measurements per patient (range 0–10 for T cells and 0–9 for B cells).

The results of the immunological investigations are summarized in **Table 4** and **Supplementary Tables 1-4**.

**Table 4:** Immunological investigations for STAT1 GOF and LOF patients summarized.

Biological investigations	Gain-of-function patients				Loss-of-function patients			
	Patients tested (n)	Normal (%)	Decreased (%)	Increased (%)	Patients tested (n)	Normal (%)	Decreased (%)	Increased (%)
IgG levels	18	16 (89%)	1 (6%)	1 (6%)	5	3 (60%)	2 (40%)	
IgA levels	18	16 (89%)	2 (11%)		5	4 (80%)	1 (20%)	
IgM levels	18	13 (72%)	5 (28%)		5	5 (100%)		
IgG1	10	8 (80%)		2 (20%)	1			1 (100%)
IgG2	10	7 (70%)	3 (30%)		1	1 (100%)		
IgG3	10	9 (90%)		1 (10%)	1	1 (100%)		
IgG4	11	4 (36%)	6 (55%)	1 (9%)	1	1 (100%)		
CD3+ T-lymphocytes	18	16 (89%)	2 (11%)		5	4 (80%)		1 (20%)
CD4+ T-lymphocytes	18	15 (83%)	2 (11%)	1 (6%)	5	5 (100%)		
CD8+ T-lymphocytes	18	16 (89%)	2 (11%)		5	4 (80%)		1 (20%)
CD19+ B-lymphocytes	18	13 (72%)	4 (22%)	1 (6%)	5	5 (100%)		
CD56+ NK-cells	18	7 (39%)	10 (56%)	1 (6%)	5	4 (80%)	1 (20%)	
CD4/CD8 ratio	17	15 (88%)	2 (12%)		5	3 (60%)	2 (40%)	
Naive B-cells	17	9 (53%)		8 (47%)	5	4 (80%		1 (20%)
IgM memory B-cells	17	11 (65%)	5 (29%)	1 (6%)	5	4 (80%)	1 (20%)	
Class-switched B-cells	17	9 (53%)	8 (47%)		5	3 (60%)	2 (40%)	
Transitory B-cells	17	11 (65%)	3 (18%)	3 (18%)	5	3 (60%)	1 (20%)	1 (20%)
Plasmablasts	17	10 (59%)	6 (35%)	1 (6%)	5	4 (80%)	1 (20%)	
CD21 low-B-cells	17	8 (47%)	6 (35%)	3 (18%)	5	4 (80%)	1 (20%)	
CD4+ memory T-cells	16	14 (88%)	2 (12%)		5	4 (80%)		1 (20%)
Follicular CD4+ T-cells	16	14 (88%)		2 (12%)	5	4 (80%)		1 (20%)
CD4+ naive T-cells	16	13 (81%)	1 (6%)	2 (13%)	5	5 (100%)		
CD4+ recent thymic emigrants	16	11 (69%)		5 (31%)	5	2 (40%)	3 (60%)	
Double negative T-cells	16	8 (50%)	2 (13%)	6 (38%)	5	1 (20%)	4 (80%)	
CD8+ naive T-cells	16	13 (81%)	1 (6%)	2 (13%)	5	3 (60%)	2 (40%)	
Early CD8+ effector/memory T-cells	16	9 (56%)	1 (6%)	6 (38%)	5	3 (60%)	1 (20%)	1 (20%)
Late CD8+ effector/memory T-cells	16	12 (75%)	3 (19%)	1 (6%)	5	4 (80%)		1 (20%)
Regulatory T-cells	16	12 (75%)	2 (13%)	2 (13%)	5	2 (40%)		3 (60%)
CD4+ Th17 (IL-17+) T-cells	14	6 (43%)	8 (57%)		4	3 (75%)	1 (25%)	
Stimulation index for PHA	7	7 (100%)			1	1 (100%)		
T-cell receptor repertoar	2	2 (100%)			1	1 (100%)		
NK-cell functional assay	11	11 (100%)			3	3 (100%)		
CD4 Th1 (IFN-gamma+) T-cells	14	11 (79%)	2 (14%)	1 (7%)	5	2 (40%)	1 (20%)	2 (40%)

Results denominated as normal, increased or decreased according to age-adjusted reference values. See **Supplementary Tables 1–4** for detailed immunological results in each of the patients.

#### 3.5.1 Immunological findings in GOF patients (n=18)

Immunoglobulins (IgG, IgA and IgM) were within the reference range in 61% (11/18) of patients. One patient had decreased levels of all immunoglobulin classes and received immunoglobulin substitution treatment. IgG4 was reduced in 55% (6/11) and IgG2 reduced in 30% (3/10) of patients.

NK-cell lymphopenia was found in 56% (10/18) and B-cell lymphopenia in 22% (4/18) of patients. One patient had reduced numbers of all lymphocyte subsets.

Deviating B-cell subpopulations were observed in 88% (15/17) of the patients, with an increased proportion of naïve B cells and a decreased proportion of class-switched B cells observed in 47% (8/17). Among the T cell subpopulations, 38% (6/16) had increased percentages of CD4-CD8- double-negative T cells (DNTs).

The percentage of Th17 cells was reduced on one or more measurements in 57% (8/14) of the patients. In patients with serial measurements (n=9), the values fluctuated from below the normal range to within the normal range in 33% of patients. The percentage of Th17 cells increased after the initiation of treatment with JAKi (n=5).

A systematic functional NK-cell degranulation assay was performed in 11 patients, with quantitative flow cytometric analysis of changes in surface-expression of CD107a after exposure to K562 cells. The assay results were normal in all patients tested.

Lymphocyte proliferation upon stimulation with phytohemagglutinin (PHA) was normal in all tested patients (n=6).

#### 3.5.2 Immunological findings in LOF patients (n=5)

Reduced levels of IgG were observed in 40% (2/5) of the patients, none of whom received immunoglobulin substitution treatment.

Lymphocyte quantification was normal in 60% (3/5) of patients. The CD4+/CD8+ T cell ratio was reduced in 40% (2/5) of patients.

Lymphocyte proliferation upon stimulation with PHA was normal in the one patient tested.

### 3.6 Treatment

#### 3.6.1 Treatment in STAT1 GOF patients

##### 3.6.1.1 Antifungals

All the patients used antifungals, either on-demand or long-term prophylactic treatment (reported in 100% and 41%, respectively). Fluconazole was the antifungal most commonly used, in 94% (17/18) of patients. Fluconazole resistance was detected in one patient, but the patient responded well to voriconazole. Amphotericin B for suspected Aspergillus infection was used in one patient.

##### 3.6.1.2 JAK inhibitors

JAKi were used in 28% (5/18) of STAT1 GOF patients, including one child. Barcitinib was used in three, and tofacitinib and ruxolitinib were used in two patients. Two patients were sequentially treated with two different JAKi due to the unsatisfactory effect of the first treatment.

The indications for starting JAKi were CMC, autoimmunity and gastrointestinal problems (i.e. esophageal inflammation, strictures and ulcers, inflammatory enteropathy). JAKi was used as a bridge-to-transplant in one patient with severe immune dysregulation and combined immunodeficiency. Decisions on initiating JAKi treatment were done on an individual basis. Clinical improvement was observed in 80% (4/5) of patients. One patient had no response to treatment and another had a declining response over time.

The daily dose administered of Tofacitinib was 10 mg (corresponding to 4,8 and 6,1 mg/m2/d), while the daily dose for Barcitinib was 4mg (corresponding to 2,3 and 2,4mg/m2/d). The pediatric patient was treated with Ruxolitinib at a dose of 10mg/m2/d. The daily dose of Tofacitinib and Ruxolitinib was divided in two equal doses, while Barcitinib was administered once daily.

Reported side effects were peritonsillar abscess, fatigue and weight gain.

##### 3.6.1.3 Other immunosuppressants

Rituximab was effective in treating AIHA and autoimmune hypertriglyceridemia. Intravenous immunoglobulins and steroids were not effective in treating AIHA in our cohort.

Other immunosuppressants used were colchicine, adalimumab, steroids, tacrolimus and mycophenolate.

##### 3.6.1.4 Stem cell transplantation

HSCT was successfully performed in one patient at the age of 9.5 years, with a 10/10 matched sibling donor. The patient’s conditioning regimen consisted of anti-thymocyte globuline (ATG), fludarabine, treosulphane and thiotepa. Graft-versus-host prophylaxis consisted of cyclosporine and mycophenolate mofetil. Two years post-SCT the patient was healthy and with good immune-reconstitution. Prior to SCT, the patient had diabetes mellitus and reduced pulmonary function, both of which persisted after SCT.

#### 3.6.2 Treatment in STAT1 LOF patients

##### 3.6.2.1 Antituberculosis treatment

Disseminated BCG infections were managed according to current national guidelines and resistance pattern related to the BCG-strain used in Norway.

##### 3.6.2.2 Antifungals

In the LOF group, 40% (2/5) of the patients used antifungals on demand.

##### 3.6.2.3 Immunosuppressive treatment

Methotrexate was successfully administered for mucocutaneous pemphigoid.

## 4 Discussion

In the present study 23 Norwegian patients with STAT1-related disease are presented, making this the largest nationwide study on STAT1 patients to date. To our best knowledge, all patients with a molecularly confirmed *STAT1*-related disease in Norway were identified. This was enabled by a well-established national immunological network and extended collaboration with the genetic departments performing *STAT1* sequencing. This provides us with a unique opportunity to estimate the prevalence of STAT1-related disease in Norway. Norway has a population of approximately 5.56 million people, giving an estimated prevalence of STAT1 related disease in Norway of 1:240 000. However, due to the broad phenotype and unawareness of the diagnosis among clinicians not familiar with IEIs, there is probably many undiagnosed patients. Thus, the true prevalence is probably higher. Nonetheless, our findings indicate that STAT1-related disease is one of the more common IEIs (14).

### 4.1 STAT1 GOF patients

CMC was the most consistent clinical feature in the STAT1 GOF patients (94%), as was increased susceptibility to infections in general, consistent with previous reports (7).

Increased morbidity due to herpesviruses was observed, consistent with previous findings (9). Severe ulcerative esophagitis due to CMV was observed in one patient and necessitated parenteral nutrition. Reactivation of VZV caused severe disease in 11% of patients, including VZV meningitis and retinitis, and around 30% suffered herpes zoster. As a measure of the immune system’s capacity to handle herpesviruses patients are routinely asked about the clinical course of their primary varicella infection. Importantly, our findings show that a mild primary infection does not exclude the possibility of severe morbidity from herpesvirus later in life.

Autoimmune phenomena are well documented in STAT1 GOF and were seen in >50% of our patients. As reported by Strøm (15), one of our patients adds a new form of autoimmunity to STAT1 GOF, namely autoimmune hypertriglyceridemia due to GPIHBP1 autoantibodies. Autoimmune hypertriglyceridemia has been found in a limited number of patients (16). It seems reasonable to screen for hyperlipidemia in the routine follow-up of STAT1 patients and to consider autoimmune hypertriglyceridemia in cases of an unexplained hypertriglyceridemia.

Severe ophthalmic manifestations were seen in 50% of our STAT1 GOF patients. Ophthalmologic complications are sparsely documented in STAT1 GOF (7), ophthalmic health is in fact not mentioned at all in some newer review papers (17, 18). Our findings suggest that ophthalmic complications are underreported in STAT1 GOF, but are of major impact for the affected patients. When following STAT1 patients, it is important to ask about eye health in the routine anamnesis. To prevent chronic eye damage, these patients require regular ophthalmologic follow-up.

Inflammatory dermal and ophthalmic manifestations due to Demodex mites are increasingly being reported in IEIs like STAT1 GOF (19–22). Demodex mites were found to be the causative agent for a pustular skin infection in one of our patient. Systematic search for Demodex were not undertaken in the other patients suffering dermal and ophthalmic manifestations. There is a well-established association between Demodex and rosacea (23), and Demodex are also believed to play a role in ocular rosacea (24). In our patient population, three patients were diagnosed with rosacea, of which two of them also had ophthalmic manifestations. Although not formally diagnosed, the patients with blepharoconjunctivitis and chalazion have symptoms compatible with ocular rosacea. Our findings add more data to the association between rosacea-like demodicosis and STAT1 GOF (20). In patients with persistent dermatological and ophthalmologic manifestations, rosacea and Demodex should be considered possible etiologies, as this may influence the choice of treatment. Rosacea and demodicosis might be underrecognized in STAT1 GOF.

### 4.2 STAT1 LOF patients

AD STAT1 LOF is associated with MSMD, but has not previously been associated with an increased susceptibility to autoimmunity, inflammation and malignancy (25). Our cohort comprise five patients with AD STAT1 LOF, of which only one of them presented with a phenotype of MSMD only. Disseminated BCG infection was diagnosed in two more patients, but these had additional heterogeneous disease manifestations.

The STAT1 LOF patients not suffering BCG infection was not vaccinated and thus were never exposed to the bacillus. STAT1 GOF have also been linked to a predisposition to mycobacterial infections (7, 9). Of note, complications secondary to BCG vaccination were not seen in any of our STAT1 GOF patients. This was despite the fact that more than 50% of the patients were vaccinated as part of the routine Childhood Immunization Program.

The authors question whether the p.Ala246Thr variant might be associated with a more severe and multifaceted phenotype. Our two patients had autoimmune, inflammatory and oncological manifestations, which up until now have not been associated with STAT1 LOF (9, 12, 25). Both patients had hematological malignancies, which was also reported by Chen et al. in one patient with the same mutation (26).

One of our patients had congenital heart disease, similarly to what has been previously reported in another STAT1 LOF patient (26). Our findings add more data to the speculation of whether there is an association between STAT1 LOF and congenital malformations.

### 4.3 Immunological findings

Broad immunological investigations were performed in our patient cohort, with various immunological deviations observed. However, there are no pathognomonic tests for STAT1, and genetic testing is necessary to diagnose STAT1-related disease.

The most consistent immunological abnormality previously reported in STAT1 GOF is a reduced frequency of Th17-cells (9). This can, however, also be found in other conditions associated with CMC and is not pathognomonic for STAT1 GOF (27). Reduced levels of Th17 cells were found in 57% of STAT1 GOF patients. However, the values fluctuated over time, from below to within the normal range. The measurement of Th17 cells cannot be considered a robust assay to screen for STAT1 GOF, and a normal Th17 value cannot be used to exclude the possibility of STAT1 GOF.

The immunological background for the increased autoimmunity in STAT1 GOF is not fully understood but is believed to be caused by increased responses to type I IFN signaling (3, 7). Approximately 40% of our patients had increased levels of DNTs. Decreased percentages of regulatory T cells were seen in 13% (2/16) of the patients, one of whom had an IPEX-like (Immune dysregulation, polyendocrinopathy, enteropathy X-linked) phenotype. Deviations in DNTs and regulatory T cells have previously not been associated with STAT1 GOF (28, 29).

Altered humoral immunity has been reported in STAT1 GOF (7, 30, 31). The present study add additional data to this matter, as 50% of our patients had deviations in B-cell subpopulations and 22% had B cell lymphopenia.

NK-cell lymphopenia is well documented in STAT1 GOF (2, 7, 9). Compared to previous findings, a greater proportion of our patients (60%) had decreased numbers of NK-cells. However, none of our patients had pathologic NK-cell functional assays, contrary to previous findings (32).

### 4.4 Summary

The present study of STAT1-related disease in Norway adds novel clinical characteristics to STAT1 GOF, making an already broad phenotype even broader. The surprising finding of troublesome ophthalmic manifestations in 50% of patients should be considered, as this warrants specialized treatment and follow-up.

Our findings strongly support the assumption that STAT1 LOF may be a more complex condition than originally thought (26). A multidisciplinary approach is warranted for both STAT1 GOF and LOF patients, and one must be aware of the possibility of malignancies in STAT1 LOF.

## Data Availability

The datasets presented in this article are not readily available because according to Norwegian GDP-rules, clinical data and lab results cannot be made publicly available. Immunological assays were done as part of routine follow-up of patients, and are part of electronic patient records. Requests to access the datasets should be directed to the corresponding author.
