# Supplementary material for: A Norwegian cohort with STAT1-related disease – further expanding the clinical phenotype
Source: Front Immunol. 2025 Aug 13;16:1620291. doi: 10.3389/fimmu.2025.1620291 (PMC12381566; doi:10.3389/fimmu.2025.1620291)
Supplement: Supplementary file 1 [file DataSheet1.pdf]

Patient	Age at sampling (years)	CD4+ Th-17 (IL17+) T-cells (reference range 1,20 - 4,10%)
P1	18	Decreased (0,90%)
	18	Normal (1,2%)
	18	Normal (1,2%)
	19	Decreased (0,80%)
	20	Normal (1,4%)
	21	Decreased (0,93%)
P2	15	Decreased (0,56%)
P3	8	Normal (1,32%)
P4	34	Decreased (0,89%)
	37	Decreased (0,48%)
	39	Decreased (0,83%
	41	Decreased (0,84%)
P5	38	Decreased (0,51)
	40	Decreased (0,60)
	44	Decreased (1,13)
P6	30	Normal (1,35%)
	30	Decreased (0,56%)
	32	Decreased (1,19)
	34	Normal (1,52%)
	34	Decreased (1,09%)
	34	Decreased (0,80%)
	35	Normal (1,4%)
P7		N/A
P8	14	Normal (1,4%)
	14	Normal (2,44%)
	16	Normal (2,8%)
P11	3	Decreased (0,24%)
	3	Decreased (0,23%)
	10	Decreased (1,17%)
P12	1	Decreased (0,22)
	4	Decreased (0,69%)
	5	Decreased (0,86%)
P13	56	Normal (1,29%)
P14		N/A
P15	30	Decreased (0,6%)
P16	44	Normal (1,29%)
	47	Normal (1,55%)
P19	40	Normal (1,71%)
	41	Normal (1,6%)
	42	Normal (2,1%)
P20		N/A
P21	39	Normal (1,8%)
	40	Normal (1,34%)
P24		N/A

S1: Table showing an overview of Th17-cell measurements in STAT1-GOF patients over time. Results reported as normal or decreased, according to age-adjusted reference values. The percentage of Th17-cells in relation to CD4+ T-cells in brackets.

Family number	Patient number:	Age at sampling (years)	CD3+ T-lymphocytes (x 10E6L)	CD4+ T-lymphocytes (x 10E6L)	CD8+ T-lymphocytes (x 10E6L)	CD19+ B-lymphocytes (x 10E6L)	NK-cells (x 10E6L)	CD4/CD8 ratio (normal >1)
	**GOF-patients**							
1 p.(Glu705 Gln)	**P1:**	10	Normal (1544)	Normal (591)	Normal (514)	Normal (214)	Normal (239)	Normal
		11	Normal (968)	Normal (455)	Normal (361)	Decreased (172)	Normal (180)	Normal
		12	Normal (1115)	Normal (467)	Normal (436)	Decreased (190)	Normal (240)	Normal
		15	Normal (1052)	Normal (531)	Normal (433)	Decreased (194)	Normal (132)	Normal
		16	Normal (1172)	Normal (553)	Normal (498)	Decreased (106)	Normal (235)	Normal
		16	Normal (992)	Decreased (428)	Normal (470)	Decreased (117)	Normal (214)	Decreased (0,91)
		17	Normal (1028)	Normal (541)	Normal (393)	Decreased (92)	Decreased (93)	Normal
		17	Decreased (746)	Decreased (364)	Normal (297)	Decreased (89)	Normal (129)	Normal
		17	Normal (907)	Decreased (433)	Normal (391)	Normal (108)	Decreased (64)	Normal
		18	Normal (946)	Decreased (444)	Normal (388)	Decreased (92)	Normal (103)	Normal
		18	Normal (980)	Decreased (440)	Normal (398)	Normal (118)	Normal (104)	Normal
		18	Normal (1295)	Normal (561)	Normal (550)	Normal (121)	Normal (123)	Normal
		19	Normal (1651)	Normal (759)	Normal (791)	Normal (136)	Decreased (62)	Decreased (0,96)
		19	Normal (1047)	Decreased (386)	Normal (526)	Decreased (51)	Decreased (83)	Decreased (0,73)
		20	Normal (1989)	Normal (736)	Increased (1002)	Decreased (88)	Decreased (83)	Decreased (0,73)
		20	Normal (1570)	Normal (709)	Normal (785)	Decreased (54)	Decreased (28)	Decreased (0,90)
		20	Normal (1597)	Normal (697)	Normal (769)	Decreased (40)	Decreased (25)	Decreased (0,91)
2 p.(Arg274 Trp)	**P2:**	5	Normal (1526)	Normal (867)	Normal (521)	Normal (1174)	Decreased (60)	Normal
		7	Normal (1084)	Normal (586)	Normal (408)	Normal (861)	Decreased (40)	Normal
		10	Normal (1130)	Normal (599)	Normal (444)	Normal (552)	Decreased (47)	Normal
		11	Normal (966)	Normal (536)	Normal (365)	Normal (504)	Decreased (66)	Normal
		13	Normal (974)	Normal (527)	Normal (377)	Normal (484)	Decreased (31)	Normal
		14	Normal (1157)	Normal (570)	Normal (443)	Normal (494)	Decreased (62)	Normal
		15	Normal (1021)	Normal (548)	Normal (367)	Normal (426)	Decreased (29)	Normal
	**P3:**	0	Normal (2884)	Normal (1807)	Normal (917)	Normal (2176)	Normal (569)	Normal
		3	Normal (1353)	Normal (851)	Normal (403)	Normal (1057)	Decreased (58)	Normal

		4	Normal (1414)	Normal (774)	Normal (537)	Normal (941)	Normal (262)	Normal
		6	Normal (929)	Normal (565)	Decreased (290)	Normal (513)	Normal (103)	Normal
		7	Normal (987)	Normal (568)	Normal (313)	Normal (495)	Normal (118)	Normal
		8	Normal (775)	Normal (478)	Decreased (226)	Normal (441)	Decreased (44)	Normal
	**P4:**	30	Normal (1227)	Normal (672)	Normal (504)	Normal (457)	Decreased (67)	Normal
		31	Decreased (711)	Decreased (390)	Normal (302)	Normal (254)	Decreased (65)	Normal
		33	Normal (962)	Decreased (462)	Normal (462)	Normal (247)	Normal (124)	Decreased (1)
		37	Normal (858)	Decreased (496)	Normal (330)	Normal (268)	Decreased (63)	Normal
		41	Normal (888)	Normal (533)	Normal (289)	Normal (271)	Decreased (52)	Normal
	**P5:**	35	Normal (1201)	Normal (586)	Normal (578)	Normal (307)	Decreased (90)	Normal
		37	Normal (1047)	Normal (547)	Normal (457)	Normal (262)	Decreased (60)	Normal
		38	Normal (1042)	Normal (501)	Normal (501)	Normal (231)	Decreased (92)	Decreased (1)
		40	Normal (1058)	Normal (533)	Normal (506)	Normal (288)	Normal (138)	Normal
		44	Normal (913)	Decreased (474)	Normal (393)	Normal (250)	Decreased (33)	Normal
		45	Normal (1153)	Normal (636)	Normal (457)	Normal (242)	Decreased (68)	Normal
	**P6:**	23	Normal (1267)	Normal (852)	Normal (336)	Normal (367)	Decreased (90)	Normal
		24	Normal (962)	Normal (660)	Normal (226)	Normal (370)	Decreased (60)	Normal
		25	Normal (847)	Normal (583)	Normal (210)	Normal (433)	Decreased (53)	Normal
		28	Normal (971)	Normal (687)	Normal (230)	Normal (420)	Decreased (78)	Normal
		29	Normal (1125)	Normal (795)	Normal (280)	Increased (678)	Normal (122)	Normal
		30	Normal (919)	Normal (590)	Normal (281)	Increased (557)	Decreased (64)	Normal
		31	Normal (817)	Normal (584)	Decreased (185)	Increased (581)	Decreased (87)	Normal
		31	Normal (904)	Normal (656)	Decreased (197)	Increased (946)	Decreased (75)	Normal
		32	Normal (1433)	Normal (1047)	Normal (310)	Increased (1413)	Normal (125)	Normal
		33	Normal (1271)	Normal (900)	Normal (262)	Increased (1781)	Decreased (84)	Normal
		34	Decreased (725)	Decreased (454)	Decreased (196)	Increased (780)	Decreased (70)	Normal
		35	Normal (1161)	Normal (795)	Normal (275)	Decreased (<5)	Decreased (67)	Normal
		35	Normal (1349)	Normal (916)	Normal (334)	Normal (144)	Decreased (65)	Normal
3 p.(Lys388 Arg)	**P7:**	1	Normal (1748)	Normal (1219)	Normal (415)	Normal (1675)	Decreased (161)	Normal
		2	Decreased (1210)	Decreased (770)	Decreased (357)	Normal (971)	Normal (113)	Normal

		2	Normal (1221)	Normal (526)	Normal (482)	Normal (703)	Decreased (81)	Normal
		5	Normal (978)	Normal (574)	Decreased (285)	Normal (463)	Decreased (54)	Normal
		5	Normal (1102)	Normal (562)	Normal (470)	Normal (355)	Decreased (58)	Normal
		6	Normal (946)	Normal (441)	Normal (424)	Normal (217)	Decreased (50)	Normal
		6	Decreased (483)	Decreased (290)	Decreased (166)	Normal (216)	Decreased (29)	Normal
		6	Normal (813)	Normal (413)	Normal (347)	Normal (227)	Decreased (55)	Normal
		7	Normal (716)	Normal (454)	Decreased (225)	Decreased (172)	Decreased (55)	Normal
		7	Normal (993)	Normal (469)	Normal (465)	Normal (202)	Normal (113)	Normal
		9	Decreased (448)	Decreased (297)	Decreased (123)	Decreased (69)	Decreased (20)	Normal
		9	Decresed (431)	Decreased (264)	Decreased (142)	Decreased (140)	Decreased (30)	Normal
		9	Decreased (323)	Decreased (201)	Decreased (104)	Decreased (<5)	Decreased (14)	Normal
		10	Decreased (371)	Decreased (231)	Decreased (121)	Decreased (<5)	Decreased (14)	Normal
		10	Decreased (297)	Decreased (169)	Decreased (114)	Decreased (<5)	Decreased (15)	Normal
		10	Decreased (270)	Decreased (144)	Decreased (111)	Decreased (<5)	Decreased (19)	Normal
		10 (1 month post-HSCT)	Decreased (624)	Decreased (108)	Normal (493)	Decreased (<5)	Normal (232)	Decreased (0,22)
		10 (3 months post-HSCT)	Normal (1654)	Decreased (318)	Increased (1326)	Normal (345)	Normal (286)	Decreased (0,24)
		10 (6 months post-HSCT)	Normal (1990)	Normal (418)	Increased (1576)	Normal (371)	Normal (205)	Decreased (0,27)
		11 (1 year post-HSCT)	Normal (1661)	Normal (570)	Normal (1065)	Normal (411)	Normal (162)	Decreased (0,54)
		11 (1,5 year post-HSCT)	Normal (1632)	Normal (573)	Normal (1026)	Increased (676)	Normal (258)	Deceased (0,56)
4 p.(Lys350 Glu)	**P8:**	10	Normal (1357)	Normal (632)	Normal (432)	Normal (303)	Normal (160)	Normal
		11	Normal (1677)	Normal (765)	Normal (616)	Normal (306)	Normal (271)	Normal
		11	Normal (1274)	Normal (580)	Normal (429)	Normal (299)	Normal (177)	Normal
		11	Normal (1357)	Normal (615)	Normal (449)	Normal (260)	Normal (185)	Normal
		12	Normal (1339)	Normal (587)	Normal (467)	Normal (330)	Normal (174)	Normal
		12	Normal (1471)	Normal (551)	Normal (480)	Normal (314)	Normal (220)	Normal
		13	Normal (1591)	Normal (665)	Normal (556)	Normal (325)	Normal (232)	Normal
		14	Normal (1599)	Normal (676)	Normal (537)	Normal (385)	Normal (234)	Normal
		14	Normal (1202)	Normal (575)	Normal (433)	Normal (318)	Normal (141)	Normal
		15	Normal (1643)	Normal (782)	Normal (581)	Normal (514)	Normal (109)	Normal
		15	Normal (1383)	Normal (691)	Normal (495)	Normal (428)	Normal (81)	Normal

		16	Normal (1603)	Normal (721)	Normal (495)	Normal (334)	Decreased (84)	Normal
	**P16:**	44	Normal (1449)	Normal (588)	Normal (749)	Normal (106)	Normal (219)	Decreased (0,79)
		44	Normal (1675)	Normal (696)	Normal (877)	Normal (118)	Normal (236)	Decreased (0,79)
		47	Normal (1204)	Normal (583)	Normal (514)	Decreased (75)	Decreased (91)	Normal
6 p.(Ile248Asn)	**P11:**	3	Increased (5461)	Increased (3096)	Increased (2159)	Increased (2509)	Normal (777)	Normal
		3	Increased (5213)	Increased (3031)	Increased (1907)	Normal (1644)	Normal (866)	Normal
		3	Normal (4163)	Increased (2480)	Normal (1492)	Normal (1324)	Normal (670)	Normal
		4	Normal (3849)	Normal (2199)	Normal (1490)	Normal (1462)	Normal (529)	Normal
		7	Normal (3135)	Normal (1619)	Normal (1404)	Normal (985)	Normal (776)	Normal
		8	Normal (3763)	Normal (1832)	Normal (1608)	Normal (1082)	Normal (578)	Normal
		8	Normal (3532)	Normal (1842)	Normal (1429)	Normal (887)	Normal (463)	Normal
		9	Normal (3865)	Increased (2081)	Normal (1500)	Normal (931)	Normal (358)	Normal
		10	Normal (3852)	Normal (1635)	Increased (1817)	Normal (887)	Normal (454)	Decreased (0,90)
	**P12:**	1	Normal (5628)	Normal (3402)	Normal (2006)	Normal (1173)	Normal (694)	Normal
		4	Normal (3788)	Normal (1864)	Increased (1625)	Normal (903)	Normal (426)	Normal
		5	Increased (4814)	Increased (2405)	Increased (2073)	Normal (786)	Normal (347)	Normal
		6	Normal (3434)	Normal (1718)	Normal (1438)	Normal (572)	Normal (240)	Normal
	**P19:**	40	Normal (1573)	Normal (772)	Normal (807)	Normal (325)	Increased (767)	Decreased (0,96)
		42	Normal (1746)	Normal (973)	Normal (794)	Normal (421)	Increased (432)	Normal
7 p.(Ala267Val)	**P13:**	45	Normal (1253)	Normal (655)	Normal (540)	Normal (255)	Normal (118)	Normal
		51	Normal (882)	Decreased (464)	Normal (396)	Normal (177)	Decreased (81)	Normal
		55	Normal (1243)	Normal (726)	Normal (494)	Normal (316)	Normal (374)	Normal
		56	Normal (1187)	Normal (710)	Normal (433)	Normal (354)	Normal (176)	Normal
		56	Normal (1489)	Normal (938)	Normal (493)	Normal (435)	Normal (200)	Normal
		58	Normal (1510)	Normal (902)	Normal (574)	Normal (342)	Normal (167)	Normal
	**P14:**	21	Normal (1454)	Normal (677)	Normal (646)	Normal (388)	Normal (214)	Normal
		33	Normal (1046)	Normal (548)	Normal (442)	Normal (187)	Normal (157)	Normal
		34	Normal (1070)	Normal (579)	Normal (442)	Normal (162)	Normal (141)	Normal
	**P21:**	29	Normal (804)	Decreased (491)	Normal (220)	Decreased (93)	Decreased (61)	Normal

		34	Normal (1085)	Normal (608)	Normal (385)	Normal (218)	Normal (102)	Normal
		35	Normal (1028)	Normal (516)	Normal (435)	Normal (202)	Decreased (69)	Normal
		39	Normal (979)	Decreased (488)	Normal (432)	Normal (159)	Normal (140)	Normal
8 p.(Ala267 Ser)	**P15:**	30	Normal (1302)	Normal (705)	Normal (535)	Decreased (69)	Decreased (40)	Normal
10 p.(Val653I le)	**P20:**	2	Normal (n/a)	Normal (n/a)	Normal (n/a)	Normal (n/a)	Normal (n/a)	n/a
		7	Normal (n/a)	Normal (n/a)	Decreased (260)	Normal (n/a)	Decreased (47)	n/a
		8	Normal (n/a)	Normal (n/a)	Normal (615)	Normal (n/a)	Normal (149)	n/a
12 p.(Arg274 Trp)	**P24:**	40	Decreased (815)	Normal	Decreased (232)	Normal	Decreased (54)	n/a
		40	Decreased (1028)	Normal	Decreased (237)	Normal	Decreased (85)	n/a
		46	Normal (1368)	n/a	Decreased (134)	n/a	Normal (124)	n/a
	**LOF patients**							
5 p.(Asn650 Asp)	**P9:**	2	Normal (5575)	Normal (3380)	Normal (2121)	Normal (2693)	Normal (853)	Normal
		3	Increased (6369)	Increased (3239)	Increased (2895)	Normal (1513)	Increased (1348)	Normal
		3	Increased (4608)	Increased (2584)	Increased (1857)	Normal (1293)	Normal (760)	Normal
		4	Normal (3387)	Normal (1582)	Normal (1455)	Normal (758)	Normal (735)	Normal
		4	Normal (3320)	Normal (1746)	Normal (1327)	Normal (774)	Normal (397)	Normal
		5	Normal (3433)	Normal (1546)	Increased (1607)	Normal (705)	Normal (653)	Decreased (0,96)
		5	Normal (2700)	Normal (1504)	Normal (1045)	Normal (647)	Normal (320)	Normal
		6	Normal (2654)	Normal (1488)	Normal (1010)	Normal (572)	Normal (390)	Normal
		7	Normal (2835)	Normal (1566)	Normal (1106)	Normal (610)	Normal (467)	Normal
		7	Normal (3109)	Normal (1721)	Normal (1215)	Normal (634)	Normal (644)	Normal
		8	Normal (1981)	Normal (1068)	Normal (778)	Normal (333)	Normal (351)	Normal
	**P10:**	32	Increased (4803)	Normal (740)	Increased (4014)	Normal (407)	Normal (224)	Decreased (0,18)
		33	Increased (3412)	Normal (589)	Increased (2810)	Normal (340)	Normal (147)	Decreased (0,21)
		34	Increased (3151)	Normal (677)	Increased (2462)	Normal (358)	Normal (150)	Decreased (0,27)
		35	Normal (2132)	Normal (560)	Increased (1558)	Normal (245)	Normal (131)	Decreased (0,36)
		35	Normal (1316)	Decreased (382)	Normal (943)	Normal (143)	Normal (129)	Decreased (0,41)
		39	Normal (1640)	Decreased (438)	Increased (1163)	Normal (207)	Normal (134)	Decreased (0,38)
	**P17:**	54	Normal (1447)	Normal (917)	Normal (559)	Normal (201)	Normal (179)	Normal

9 p.(Ala246 Thr)	**P18:**	29	Normal (1206)	Normal (506)	Normal (734)	Normal (164)	Decreased (55)	Decreased (0,69)
11 p.(Lys637 Glu)	**P22:**	23	Normal (1238)	Normal (740)	Normal (497)	Normal (147)	Normal (209)	Normal
		31	Normal (1988)	Normal (1067)	Normal (921)	Normal (144)	Normal (301)	Normal

S2: Table showing lymphocyte quantifications over time in all the STAT1 patients. Results reported as normal (black color), increased (red color) or decreased (blue color) according to age-adjusted reference values, with the actual cell count in brackets.

**Family**	**Patient**	**Age at sampling (years)**	**CD4+ memory T-cells** (%)	**Follicular CD4+ T-cells** (%)	**CD4+ naive T-cells** (%)	**CD4+ recent thymic emigrants** (%)	**Double negative T-cells** (%)	**CD8+ naive T-cells** (%)	**Early CD8+ effector/ memory T-cells** (%)	**Late CD8+ effector/ memory T-cells** (%)	**Regulatory T-cells** (%)
	**GOF patients**										
1 p.(Glu705Gln)	**1**	17	Normal (47,5)	Normal (11,6%)	Normal (70,0%)	Normal (68,8%)	Increased (2,3%)	Normal (85,1%)	Normal (11,6%)	Normal (2,6%)	Normal (5,1%)
		18	Normal (47,4)	Normal (13,1%)	Increased (71,9%)	Normal (67,7%)	Normal (2,0%)	Increased (91,4%)	Normal (6,7%)	Decreased (0,8%)	Normal (4,7%)
		19	Decreased (46,4%)	Normal (14,7%)	Increased (75,3%)	Normal (60,8%)	Normal (1,8%)	Increased (89,6%)	Normal (8,0%)	Decreased (1,6%)	Normal (4,9%)
		19	Normal (52,2)	Normal (13,8%)	Normal (68,6%)	Normal (57,8%)	Normal(1,9%)	Increased (91,7%)	Normal (5,0%)	Decreased (1,0%)	Normal (5,2%)
		20	Decreased (46,5%)	Normal (14,9%)	Increased (72,7%)	Normal (56,3%)	Increased (2,3%)	Increased (90,1%)	Normal (6,1%)	Decreased (1,8%)	Normal (5,4%)
		20	Decreased (43,4%)	Normal (13,7%)	Increased (72,0%)	Normal (58,1%)	Increased (2,3%)	Increased (94%)	Normal (3,6%)	Decreased (0,8%)	Increased (5,9%)
2 p.(Arg274Trp)	**2**	15	Normal (33,8%)	Normal (14,7%)	Normal (72,8%)	Increased (85,5%)	Normal (3,9%)	Normal (70,0%)	Normal (6,6%)	Normal (22,6%)	Decreased (3,6%)
	**3**	8	Normal (33,4%)	Normal (11,2%)	Normal (77,0%)	Normal (73,8%)	Normal (5,0%)	Normal (70,9%)	Increased (9,7%)	Normal (18,8%)	Normal (6,7%)
	**4**	31	Normal (44,7%)	Normal (12,9%)	Normal (48,4%)	Normal (75%)	Normal (2%)	Decreased (29,3%)	Normal (16,6%)	Increased (50,5%)	Normal (2,9%)
		33	Normal (44%)	Increased (14%)	Normal (59%)	Normal (57%)	Increased (2,3%)	Decreased (27%)	Increased (21%)	Increased (50%)	Normal (4,7%)
		37	Normal (52,3%)	Normal (14,6%)	Normal (63,5%)	Normal (56,5%)	Increased (2,1%)	Decreased (28,1%)	Increased (17,2%)	Normal (52,2%)	Normal (3,7%)
	**5**	35	Normal (58,9%)	Increased (14,7%)	Normal (53,1%)	Normal (63,9%)	Increased (3,2%)	Normal (53,9%)	Normal (8,1%)	Normal (34,2%)	Normal (4,1%)
		38	Normal (64,4%)	Increased (19,9%)	Normal (51,0%)	Normal (58,4%)	Increased (2,4%)	Normal (44,5%)	Normal (10,9%)	Normal (40,9%)	Normal (5,1%)
		44	Normal (58,3%)	Normal (20,0%)	Normal (56,3%)	Normal (60,5%)	Increased (2,6%)	Normal (47,7%)	Normal (9,8%)	Normal (38,2%)	Normal (3,4%)
	**6**	24	Normal (50,7%)	Increased (14,3%)	Normal (51,6%)	Normal (76,4%)	Increased (3,5%)	Normal (66,1%)	Normal (15,9%)	Normal (13,7%)	Normal (2,9%)
		25	Normal (52%)	Increased (17%)	Normal (53%)	Normal (69%)	Increased (5,4%)	Normal (82%)	Normal (16%)	Normal (6,8%)	Increased (6,9%)
		28	Normal (63,2%)	Normal (16,6%)	Normal (58,8%)	Normal (62,0%)	Increased (3,9%)	Normal (73,4%)	Normal (13,8%)	Normal (11,6%)	Normal (3,9%)
		31	Normal (63,7%)	Increased (18,0%)	Normal (60,9%)	Normal (60,1%)	Normal (1,5%)	Normal (62,8%)	Normal (15,5%)	Normal (20,1%)	Normal (5,3%)
		31	Normal (51,5%)	Increased (18,8%)	Normal (62,3%)	Normal (62,2%)	Increased (3,6%)	Normal (77,7%)	Normal (8,0%)	Normal (13,1%)	Normal (5,6%)
		32	Normal (48,5%)	Increased (19,6%)	Normal (63,6%)	Normal (58,7%)	Increased (3,5%)	Normal (79,7%)	Normal (5,6%)	Normal (13,1%)	Normal (5,1%)
		33	Normal (47,1%)	Normal (15,5%)	Increased (74%)	Normal (53,5%)	Increased (7,7%)	Normal (84,7%)	Normal (6,6%)	Normal (7,4%)	Increased (6,8%)
		34	Normal (56,9%)	Normal (20,4%)	Normal (56,9%)	Normal (55,1%)	Increased (6,7%)	Normal (77,7%)	Normal (11,7%)	Normal (9,1%)	Normal (4,9%)
		35	Normal (62,3%)	Increased (30,4%)	Normal (59,5%)	Normal (52,5%)	Increased (3,8%)	Normal (74,0%)	Normal (13,2%)	Normal (10,0%)	Normal (4,3%)
		35	Normal (59,5%)	Increased (24,8%)	Normal (57,6%)	Normal (56,1%)	Increased (2,6%)	Normal (60,8%)	Normal (12,5%)	Normal (24,6%)	Normal (4,7%)
3 p.(Lys388Arg)	**7**	9	Normal (74,7%)	Normal (24,2%)	Decreased (41,3%)	Normal (72,9%)	Normal (5,3%)	Normal (58,1%)	Increased (21,1%)	Normal (18,8%)	Decreased (2,2%)
		9	Normal (75,6%)	Normal (23,9%)	Decreased (38,7%)	Normal (67,8%)	Normal (5,2%)	Normal (46,1%)	Increased (31,1%)	Normal (20,8%)	Decreased 2,0%)
		10	Normal (78%)	Normal (25,3%)	Normal (46,1%)	Normal (78,3%)	Normal (3,9%)	Normal (66,9%)	Increased (15,5%)	Normal (9,8%)	Decreased (2,7%)
4 p.(Lys350Glu)	**8**	10	Normal (49,9%)	Decreased (6,6%)	Normal (60,3%)	Increased (85,7%)	Normal (2,4%)	Normal (77,1%)	Normal (13,2%)	Normal (9,4%)	Normal (5,7%)
		12	Normal (54,3%)	Normal (9,0%)	Normal (58,5%)	Increased (86,7%)	Normal (3,1%)	Normal (81,3%)	Normal (12,7%)	Normal (5,7%)	Normal (6,5%)

		12	Normal (49,8%)	Normal (7,6%)	Normal (56,8%)	Increased (89,1%	Normal (3,9%)	Normal (71,4%)	Increased (18,3%)	Normal (9,8%)	Normal (7,7%)
		14	Normal (52,5%)	Normal (6,5%)	Normal (61,4%)	Increased (81,6%)	Normal (4,0%)	Normal (75,0%)	Normal (14,5%)	Normal (10,0%)	Normal (6,2%)
		14	Normal (55,9%)	Normal (7,4%)	Normal (60,0%)	Normal (78,9%)	Normal (3,6%)	Normal (82,6%)	Normal (9,7%)	Normal (7,0%)	Normal (6,1%)
		16	Normal (53,9%)	Normal (11,0%)	Normal (53,4%)	Increased (84,0%)	Increased (3,7%)	Normal (82,4%)	Normal (10,6%)	Normal (6,3%)	Increased (6,6%)
	**16**	44	Normal (75,4%)	Normal (14,9%)	Normal (36,4%)	Normal 63,8%)	Normal (1,2%)	Decreased (30,6%)	Increased (19,0%)	Normal (49,7%)	Normal (3,8%)
		47	Normal (78,1%)	Normal (16,5%)	Normal (41,7%)	Normal (66,4%)	Normal (1,3%)	Normal (38,1%)	Increased (18,9%)	Normal (42,3%)	Normal (3,3%)
6 p.(Ile248Asn)	**11**	3	Normal (28,0%)	Normal (7,7%)	Normal (86,0%)	Normal (86,3)	Normal (1,0%)	Normal (75%)	Increased (12,0%)	Normal (13%)	Normal (5,4%)
		3	Normal (32,0%)	Normal (9,6%)	Normal (83,5%)	Normal (90,3)	Decreased (0,9%)	Normal (65,2%)	Increased (16,8%)	Normal (17,4%)	Normal (5,2%)
		3	Normal (34,7%)	Normal (6,8%)	Normal (89,6%)	Normal (90,2)	Decreased (0,8%)	Normal (76%)	Normal (8,7%)	Normal (14,9%)	Normal (5,3%)
		9	Normal (24,6%)	Normal (8,5%)	Normal (82,2%)	Increased (87,2%)	Decreased (0,5%)	Normal (58%)	Increased (8,9%)	Normal (32,1%)	Normal (7,2%)
		10	Normal (32,4%)	Normal (10,6%	Normal (76,4%)	Increased (85,5%)	Decreased (0,3%)	Normal (42,2%)	Increased (10,7%)	Normal (45,8%)	Normal (7,4%)
	**12**	1	Normal (12%)	Decreased (2,5%)	Normal (92%)	Increased (92%)	Normal (0,8)	Normal (88%)	Normal (12,0)	Decreased (0,1%)	Normal (6,8%)
		4	Normal (28,7%)	Normal (9,8%)	Normal (83,7%)	Normal (83,9)	Decreased (0,9%)	Normal (62%)	Increased (13,7%)	Normal (24%)	Normal (6,6%)
		5	Normal (22,6%)	Normal (7,2%)	Normal (83,8%)	Normal (87,7)	Decreased (1,0%)	Normal (80,3%)	Normal (8,2)	Normal (11,1%)	Normal (7,8%)
		6	Normal (26,4%)	Normal (9%)	Normal (79,1%)	Increased (87,9%)	Normal (0,9)	Normal (70%)	Increased (11,6%)	Normal (17,4%)	Normal (8,3%)
	**19**	40	Normal (56,3%)	Normal (11,1%)	Normal (60,3%)	Normal (44,6%)	Decreased (0,2%)	Decreased (31,9%)	Normal (3,8%)	Increased (61,8%)	Increased (7,4%)
		42	Normal (54,8%)	Normal (13,9%)	Normal (63,6%)	Normal (42,1%)	Decreased (0,2%)	Normal (41,5%)	Decreased (2,6%)	Normal (53,3%)	Increased (7,6%)
7 p.(Ala267Val)	**13**	51	Increased (91,5%)	Normal (11,6%)	Normal (57,6%)	Normal (70,5%)	Normal (1,8%)	Normal (86,4%)	Normal (11,2%)	Decreased (1,1%)	Normal (3,7%)
		55	Normal (48,1%)	Normal (13,4%)	Normal (62,8%)	Normal (68,2%)	Normal (1,8%)	Normal (86,6%)	Normal (9,7%)	Decreased (2,1%)	Normal (3,9%)
		56	Normal (59,4%)	Increased (21,4%)	Normal (55,9%)	Normal (67,3%)	Normal (1,7%)	Increased (88,1%)	Normal (7,3%)	Normal (2,7%)	Normal (5,7%)
		56	Normal (63,6%)	Increased (21,6%)	Normal (50,7%)	Normal (65,1%)	Normal (1,9%)	Increased (87,2%)	Normal (8,5%)	Decreased (2,2%)	Normal (4,9%)
		58	Normal (52,3%)	Normal (17,7)	Normal (58,6%)	Normal (70,4%)	Normal (1,3%)	Normal (84,2)	Normal (12,1%)	Decreased (2,1%)	Normal (5,1%)
	**14**	33	Normal (57,4%)	Normal (12,9%)	Normal (56,6%)	Normal (70,1%)	Increased (3,0%)	Increased (88,6%)	Decreased (1,6%)	Decreased (0,7%)	Increased (8,7%)
	**21**	35	Normal (69,8%)	Normal (16m7%)	Normal (32,4%)	Normal (62,6%)	Increased (6,8%)	Normal (51,6%)	Normal (15,5%)	Normal (29,6%)	Normal (5,2%)
		39	Normal (81,5%)	Normal (16,4%)	Normal (35%)	Normal (50,8%)	Increased (7,4%)	Normal (48%)	Normal (10%)	Normal (36,9%)	Increased (6,3%)
8 p.(Ala267Ser)	**15**	30	Decreased (29,0%)	Normal (13,4%)	Increased (73,1%)	Increased (79,3%)	Normal (1,1%)	Normal (50,5%)	Normal (11%)	Normal (38,2%)	Normal (2,8%)
10 p.(Val653Ile)	**20**		Never measured								
12 p.(Arg274Trp)	**24**	52	Normal (n/a)	Normal (n/a)	Normal (n/a)	Normal (n/a)	Normal (n/a)	Normal (n/a)	Normal (n/a)	Normal (n/a)	Normal (n/a)
	**LOF patients**										
5 p.(Asn650Asp)	**9**	3	Normal (24,8%)	Normal (6,1%)	Normal (85,9%)	Normal (68,8%)	Decreased (0,6%)	Normal (40,1%)	Increased (20,1%)	Normal (38,7%)	Normal (6,5%)
		3	Normal (22,3%)	Decreased (5,1%)	Normal (86,6%)	Normal (69,1%)	Decreased (0,4%)	Normal (44,1%)	Increased (19,3%)	Normal (35,1%)	Normal (6,6%)

		4	Normal (25,8%)	Normal (6,5%)	Normal (86,6%)	Normal (68,1%)	Decreased (0,4%)	Normal (43,7%)	Increased (18,6%)	Normal (36,2%)	Normal (6,3%)
		4	Normal (21,8%)	Decreased (4,8%)	Normal (83,6%)	Normal (68,6%)	Decreased (0,3%)	Normal (44,1%)	Increased (20,0%)	Normal (33,5%)	Normal (6%)
		5	Normal (27%)	Normal (5,2%)	Normal (84,2%)	Normal (62,9%)	Decreased (0,3%)	Normal (39,6%)	Increased (23,1%)	Normal (34,9%)	Normal (5,3%)
		6	Normal (25,5%)	Normal (6,7%)	Normal (85,1%)	Normal (64,5%)	Decreased (0,3%)	Normal (52,9%)	Increased (19,0%)	Normal (26%)	Normal (5,2%)
		7	Normal (31%)	Normal (8,2%)	Normal (83%)	Normal (61,6%)	Decreased (0,4%)	Normal (50,6%)	Increased (16,2%)	Normal (30,7%)	Normal (4,6%)
		7	Normal (31,2%)	Normal (7,4%)	Normal (79,6%)	Normal (61%)	Decreased (0,3%)	Normal (44,3%)	Increased (17,7%)	Normal (34,9%)	Normal (5%)
		8	Normal (27,5%)	Normal (8,2%)	Normal (81,3%)	Normal (61,9%)	Decreased (0,3%)	Normal (50,3%)	Increased (15,9%)	Normal (31,3%)	Normal (4,6%)
	**10**	32	Normal (64,5%)	Normal (15,5%)	Normal (60,8%)	Decreased (21,1%)	Decreased (0,2%)	Decreased (16,7%)	Normal (12,7%)	Increased (67,6%)	Increased (6,0%)
		39	Normal (72,5%)	Increased (22,4%)	Normal (53,9%)	Decreased (17,8%)	Decreased (0,3%)	Decreased (30,7%)	Normal (7,6%)	Normal (55%)	Increased (7,7%)
9 p.(Ala246Thr)	**17**	54	Increased (83,3%)	Increased (33,2%)	Normal (40,6%)	Decreased (10,1%)	Decreased (0,2%)	Normal (61,8%)	Normal (3,5%)	Normal (19,2%)	Increased (6,6%)
	**18**	29	Normal (69,2%)	Normal (18,5%)	Normal (49,4%)	Decreased (4,9%)	Decreased (0,3%)	Decreased (19,6%)	Decreased (1,2%)	Normal (37,9%)	Normal (3,2%)
11 p.(Lys637Glu)	**22**	23	Normal (51,7%)	Normal (8,6%)	Normal (56%)	Normal (59%)	Normal (0,9%)	Normal (55%)	Normal (7%)	Normal (32%)	Increased (9,5%)
		31	Normal (56,2%)	Normal (9,8%)	Normal (62,8%)	Normal (51,7%)	Normal (1,4%)	Normal (51,4%)	Normal (6%)	Normal (37,7%)	Increased (8,3%)

S3: Table showing T-cell subpopulations measured over time in the STAT1-patients. Results reported as normal (black color), increased (red color) or decreased (blue color) according to age-adjusted reference values, with percentages in brackets.

Family	Patient	Age at sampling (years)	Naive B-cells (%)	IgM memory B-cells (%)	Class-switched B-cells (%)	Transitional B-cells (%)	Plasmablasts (%)	CD21 low B-cells (%)
	GOF patients							
1 p.(Glu705Gln)	1	17	Increased (85,8%)	Normal (4,8%)	Normal (5,7%)	Normal (3,1%)	Normal (1,2%)	Normal (2,3%)
		18	Normal (82,2%)	Normal (9,6%)	Normal (6,8%)	Normal (1,8%)	Normal (3,4%)	Normal (7,5%)
		19	Normal (80,7%)	Normal (10,6%)	Normal (7,1%)	Normal (0,9%)	Normal (1,6%)	Normal (1,9%)
		20	Normal (56,5%)	Normal (16,4%)	Normal (21,5%)	Increased (4,9%)	Increased (11,6%)	Normal (6,0%)
2 p.(Arg274Trp)	2	15	Increased (92,8%)	Decreased (4,3%)	Decreased (1,3%	Normal (2,8%)	Decreased (0,2%)	Decreased (2,4%)
	3	8	Increased (91,6%)	Decreased (2,1%)	Decreased (4,5%)	Increased (8,9%)	Normal (2,3%)	Decreased (1,1%)
	4	31	Increased (82,5%)	Normal (7,7%)	Decreased (0,8%)	Normal (1,6%)	Decreased (0,2%)	Normal (3,9%)
		33	Increased (83,8%)	Decreased (4,4%)	Decreased ((5,4%)	Normal (3,4%)	Decreased (0,3%)	Normal (3,5%)
	5	35	Normal (70,5%)	Normal (14,4%)	Decreased (4,5%)	Normal (0,7%)	Decreased (0,1%)	Normal (4,7%)
		44	Normal (60,3%)	Increased (26,7%)	Normal (6,0%)	Normal (1,3%)	Decreased (0,2%)	Increased (19,2%)
	6	24	Decreased (41,5%)	Normal (26,3%)	Decreased (0,4%)	Normal (0,7%)	Decreased (0,2%)	Increased (13,6%)
		25	Normal (46%)	Increased (34%)	Decreased (0,3%)	Decreased (0,2%)	Decreased (0,06%)	Increased (26%)
		28	Normal (57,2%)	Increased (41,4%)	Decreased (0,3%)	Normal (1,4%)	Decreased (0,0%)	Increased (26,7%)
3 p.(Lys388Arg)	7	9	Inreased (98,4%)	Decreased (0,9%)	Decreased (0,2%)	Increased (82,8%)	Decreased (0,1%)	Decreased (1,5%)
		9	Increased (98,2%)	Decreased (1,1%)	Decreased (0,0%)	Increased (78,8%)	Decreased (0,0%)	Decreased (0,1%)
4 p.(Lys350Glu)	8	13	Increased (89,8%)	Normal (4,6%)	Decreased (2,6%)	Normal (2,3%)	Decreased (0,2%)	Decreased (0,9%)
		14	Increased (90,4%)	Decreased (3,8%)	Decreased (3,5%)	Normal (4,8%)	Decreased (0,5%)	Decreased (1,4%)
		14	Increased (90%)	Decreased (3,9%)	Decreased (3,9%)	Normal (6,9%)	Normal (0,9%)	Decreased (1,4%)
		16	Increased (87,3%)	Normal (4,0%)	Normal (5,9%)	Normal (3,6%)	Normal (2,1%)	Normal (1,5%)
	16	44	Normal (70,7%)	Normal (18,2%)	Normal (9,3%)	Normal (2,4%)	Normal (1,2%)	Normal (3,2%)
		47	Normal (69,6%)	Normal (12,8%)	Normal (9,4%)	Normal (2,7%)	Normal (0,6%)	Normal (2,6%)
6 p.(Ile248Asn)	11	9	Increased (80,6%)	Normal (7,6%)	Decreased (6,0%)	Decreased (1,7%)	Decreased (0,2%)	Decreased (1,7%)
		10	Increased (80,7)	Normal (11,5%)	Decreased (5,0%)	Normal (6,0%)	Decreased (0,1%)	Decreased (2,1%)
	12	1	Decreased (87,3%)	Increased (7,5%)	Normal (3,6%)	Decreased (3,6%)	Normal (0,9%)	Normal (1,7%)
		4	Normal (79,7%)	Normal (10,8%)	Normal (6,1%)	Decreased (2,9%)	Decreased (0,4%)	Normal (3,7%)
		5	Normal (69%)	Normal (11,3%)	Normal (11,5%)	Decreased (1,7%)	Decreased (0,5%)	Normal (3,1%)
		6	Normal (70%)	Normal (17,8%)	Decreased (8,8%)	Normal (6,2%)	Normal (1,1%)	Normal (4,4%)
	19	40	Normal (55,8%)	Normal (19,2%)	Normal (15,8%)	Normal (0,8%)	Normal (0,5%)	Normal (3,3%)
		42	Normal (59,7%)	Normal (18,4%)	Normal (16,3%)	Normal (1,6%)	Normal (0,5%)	Normal (3,1%)
7 p.(Ala267Val)	13	51	Normal (80,1%)	Normal (10%)	Normal (4,5%)	Normal (1,3%)	Decreased (0,1%)	Normal (2,8%)
		55	Increased (87,9%)	Normal (7,3%)	Decreased (3,9%)	Normal (2,3%)	Normal (0,5%)	Normal (2,6%)
		56	Increased (85,3%)	Normal (8,3%)	Normal (4,8%)	Normal (2,7%)	Normal (0,7%)	Normal (2,8%)
		56	Increased (84,9%)	Normal (7,6%)	Normal (5,3%)	Normal (2,0%)	Normal (1,4%)	Normal (2,2%)
		58	Increased (84,2)	Normal (9,3%)	Decreased (4,3%)	Normal (2,6%)	Normal (0,7%)	Normal (2,1%)
	14	33	Normal (82,7%)	Normal (7,7%)	Normal (6,1%)	Increased (4,9%)	Normal (1,5%)	Decreased (0,9%)
	21	35	Increased (86%)	Decreased (2,2%)	Decreased (1,3%)	Normal (1,3%)	Normal (0,3%)	Normal (2,3%)
		39	Increased (90,7%)	Normal (5%)	Decreased (2,1%)	Normal (2,7%)	Normal (0,4%)	Normal (2,8%)
8 p.(Ala267Ser)	15	30	Normal (81,6%)	Normal (10,1%)	Normal (5,8%)	Decreased (0,6%)	Normal (0,4%)	Increased (11,9%)

10 p.(Val653Ile)	20	Never measured						
12 p.(Arg274Trp)	24	52	Normal (n/a)	Decreased (n/a)	Normal (n/a)	Normal (n/a)	Normal (n/a)	Normal (n/a)
	**LOF patients**							
5 p.(Asn650Asp)	9	3	Normal (79,2%)	Normal (12,9%)	Normal (6,2%)	Normal (8,8%)	Normal (0,9%)	Decreased (1,0%)
		4	Normal (72,1%)	Normal (14%)	Normal (10,8%)	Normal (3,8%)	Normal (0,8%)	Decreased (1,4%)
		4	Normal (72,6%)	Normal (16,3%)	Normal (8,7%)	Decreased (2,4%)	Normal (0,6%)	Decreased (1,2%)
		5	Normal (63,7%)	Increased (20,0%)	Normal (14%)	Decreased (1,5%)	Normal (2%)	Decreased (1,6%)
		5	Normal (52,1%)	Normal (12,6%)	Decreased (8,2%)	Decreased (1,9%)	Decreased (0,6%)	Decreased (1,1%)
		6	Normal (69,3%)	Normal (14%)	Normal (13,1%)	Decreased (2,3%)	Normal (0,6%)	Decreased (1,3%)
		7	Normal (70,4%)	Normal (15,4%)	Decreased (10,2%)	Decreased (3,2%)	Normal (0,7%)	Decreased (1,1%)
		7	Normal (68,3%)	Normal (18,1%)	Normal (11,2%)	Decreased (4,5%)	Normal (1,4%)	Decreased (1,4%)
		8	Normal (66,7%)	Normal (20,3%)	Decreased (9,9%)	Decreased (4,4%)	Decreased (0,4%)	Decreased (1,2%)
	10	32	Normal 73,1%)	Normal (10,1%)	Normal (14,1%)	Normal (2,7%)	Normal (1,7%)	Normal (1,8%)
		39	Normal (64,5%)	Normal (7,3%)	Normal (13,1%)	Normal (2,7%)	Normal (1,2%)	Normal (1,7%)
9 p.(Ala246Thr)	17	54	Normal (65,8%)	Normal (18,3%)	Normal (10,7%)	Normal (1,5%)	Normal (0,6%)	Normal (4%)
	18	29	Increased (95,7%)	Decreased (2,2%)	Decreased (0,4%)	Increased (8,0%)	Decreased (0,1%)	Normal (2%)
11 p.(Lys637Glu)	22	23	Normal (77,6%)	Normal (9,5%)	Normal (10,2%)	Normal (1,6%)	Normal (0,7%)	Normal (1,6%)
		31	Normal (65,5%)	Normal (16%)	Normal (12,9%)	Normal (0,9%	Normal (4,5%)	Normal (5,3%)

S4: Table showing B-cell subpopulations measured over time in the STAT1-patients. Results reported as normal (black color), increased (red color) or decreased (blue color) according to age-adjusted reference values at the time of sampling, with percentages reported in brackets.
